# Parturient Apoplexy in Cows

**Published:** 1892-01

**Authors:** D. McIntosh


### PARTURIENT APOPLEXY IN COWS.*

BY DR. D. McINTOSH.

When I first commenced to practice, I followed the rules laid down on the subject of parturient apoplexy in cows, viz.: Bleeding, giving large doses of physic, injections, hot cloths to the back and loins, and ice to the head, etc., and under this method of treatment very few of my patients recovered.

This state of affairs troubled me very much, and I determined to investigate and find out if we were right as to the nature of this very fatal disease. I read all I could find on the subject. The authors differed so much as to the nature and cause of the disease that I found very little benefit in this line. My next effort was to make post-mortem examinations of all the cases that died from this disease. The first post-mortem I made surprised me very much, as I found none of the characteristics of congestion or inflammation present.

* Read before the Illinois Veterinary Association.

At the next case which presented itself for post-mortem I had the assistance of a medical man well versed in pathology. We made a very careful examination and found no appearance of congestion or inflammation. I made several more post-mortems of cows, which died from this disease, with the same result. This led me to think we must be mistaken as to the true nature of this disease, and if so our treatment must be wrong.

It is a well established fact, that coma or delirium can occur without congestion or inflammation of the brain ; an over distended stomach, or deranged state of the nervous system, will cause it. Another fact to be considered is, that the symptoms of inflammation or congestion of the brain are very different from those of parturient apoplexy, especially in the early stages of the disease. These facts led me to believe that the disease must be of a nervous character and not congestion. Some writers of note think it is caused from derangement of the sympathetic nerve, which through its action on the blood vessels causes congestion of the brain. This last part is, I think, where the mistake has been made. The sympathetic nerve controls all hollow viscera, and has a special action on the uterus, and through its connection with the lumbar nerves deranges the muscles of the loins and posterior extremities first, gradually proceeding forward until the brain becomes affected, producing coma or delirium.

Taking this into consideration I changed the treatment to strong stimulants, and to my satisfaction the patients recovered. I have adopted this form of treatment ever since, and I never since lost a case of this so-called fatal disease. I consider it useless to torment the animal by pouring down large doses of physic. The sympathetic nerve controls secretion and execretion, and on that account the physic will not take effect until the sympathetic nerve is performing its function, and this can only be done by stimulants.

The following is the treatment I have adopted :

RECEIPT.

*Spt ether nit. fluid ounces XX.*
*Spt. ammoniæ aromatic, fluid ounces X.*

M.

Give three oz. at a dose every half hour, in one pint of cold water until five doses have been given ; then every hour until the remainder has been administered.

Also apply two lbs. mustard, made into a poultice with boiling water, along each side of the spinal column, and cover with blankets.

Keep the animal on its sternum by means of bundles of straw. She seems to rest easier in this position. Some one should be in close attendance until she is better. Usually in from eight to ten hours the animal will be able to rise. If the animal has not recovered at the end of that time it will be necessary to give her ten ounces more of the same kind of medicine. I have had to do this in a few cases.

In my opinion, the best prevention in this disease is to keep the cow in a good firm healthy state. This can be done by giving the animal good solid food and not too much of it. In the spring, when the weather is getting hot and the grass long and succulent, keep the cow off such pastures and put her in a place where she cannot get much grass, and feed with dry, solid feed, as hay, oats or corn. Soft sloppy diet is not good. If the animal is fat, soft and flabby, it would be well to give it one and a half pounds of epsom salts about ten days before calving. It is not wise to give it a few hours before parturition as it weakens the animal. This treatment I have advised for several years, I think with beneficial results.

---

